# Effect of Different Vitrification Techniques on Viability and Apoptotic Index of Domestic Cat Testicular Tissue Cells

**DOI:** 10.3390/ani13172768

**Published:** 2023-08-31

**Authors:** Julyne Vivian Guimarães de Carvalho, Airton R. B. Soares, Danuza L. Leão, Adriana N. Reis, Regiane R. Santos, Ana P. R. Rodrigues, Sheyla F. S. Domingues

**Affiliations:** 1 Laboratory of Wild Animal Biotechnology and Medicine, Faculty of Veterinary Medicine, Federal University of Pará, Castanhal 68740-970, Brazil; 2 Postgraduate Program in Animal Health and Production in the Amazon, Federal Rural University of the Amazon, Belém 66077-830, Brazil; 3 Postgraduate Program of Animal Reproduction in Amazon, Institute of Veterinary Medicine, Federal University of Pará, Castanhal 68740-970, Brazil; 4 Laboratory of Manipulation of Oocytes and Preantral Follicles (LAMOFOPA), State University of Ceara, Fortaleza 60714-903, Brazil

**Keywords:** devices, testicles, apoptosis, cryovial

## Abstract

**Simple Summary:**

The vitrification process stands out among cryopreservation methods because it is a practical and fast technique that reduces the risk of intracellular ice formation and cell damage. The present study aimed to compare three devices used for the vitrification of adult cat testicular biopsies for use in preservation programs. The efficiency of the methods was evaluated by histological and viability analysis of the testicular cells after the vitrification process. It is shown that these methods are efficient and can be considered an important tool for the genetic conservation of endangered wild cats, with the domestic cat providing an experimental model for the development of reproductive biotechnology in wild cat species.

**Abstract:**

Vitrification is essential for successful tissue cryopreservation and biobanking in wild cats. This study aimed to compare different methods of vitrification (Ovarian Tissue Cryosystem—OTC, Straws—STW, and Solid Surface vitrification—SSV) for testicular fragment vitrification in tom cats. Testicular fragments were recovered from five adult tom cats and subjected to equilibrium vitrification using different cryovials and methods under the same conditions of vitrification solutions and cryoprotectants. The efficiencies of the methods were evaluated using histological analysis of spermatogonia and Sertoli cell nuclei, seminiferous tubular basement membrane detachment, and the gonadal epithelium shrinkage score scale. Cell viability was assessed using Hoechst PI and Terminal deoxynucleotidyl transferase nick end labeling (TUNEL) assay. The results showed that OTC is an effective vitrification method for maintaining the distinction between spermatogonia and Sertoli cells. OTC was similar to the control for basal membrane detachment parameters (*p* = 0.05). Epithelial shrinkage was low in the SSV group, which showed the highest percentage of viable cells among the vitrified groups (*p* = 0.0023). The OTC and SSV vitrification methods were statistically similar in terms of the percentage of TUNEL-positive cells (*p* = 0.05). Therefore, OTC and SSV provide favorable conditions for maintaining viable cat testicular tissue cells after vitrification.

## 1. Introduction

Vitrification is an important cryopreservation biotechnique that preserves feline genetic resources [1]. Media glass state formation during vitrification aids in maintaining cell structure and function after warming [2]. This factor is responsible for preserving cell membranes and organelles by preventing mechanical damage and intracellular crystallization caused by cold [3].

Testicular somatic and gonadal cells are essential genetic resource biomaterials and are easily accessible samples owing to the routine gonadectomy of domestic cats. Because all wild felids are endangered species, according to the International Union for Conservation of Nature—IUCN, domestic cats are an experimental model for the reproductive biotechnic development of wild felids [4]. In adult animals, seminiferous tubule assessment provides the recovery of all types of germinal cells. It is an essential factor in preserving the genetic resource of pubertal males that unexpectedly die or require permanent sterilization [5].

The use of the vitrification biotechnique to preserve prepubertal testicular biopsies has been studied in several domestic species, such as pigs [6], mice [7], and dogs [8], to evaluate the viability of protocols. In domestic cats, studies have compared vitrification and slow freezing [9], the use of cryoprotectants for prepubertal cats [10], and the effects of warming temperatures [1,11] on test tissue. Recently, some results have been described for adult cat testicular fragment vitrification protocols and conditions of warming [12].

Plastic straws are commonly used in the primary vitrification technique because they are practical, less expensive, and optimize the space in a cryogenic cylinder. Plastic materials facilitate manipulation, are low-cost [13], and have been used for the vitrification and rapid freezing of human semen [14], embryo vitrification [15], and testicular biopsy vitrification [16]. Alternatively, Solid Surface Vitrification (SSV) provides higher cooling rates due to open aluminum cubes placed in a styrofoam box and submerged in liquid nitrogen [17]. SSV is an efficient method for the vitrification of testicular biopsies in immature rats [18] and prepubertal domestic cats [10].

The Ovarian Tissue Cryosystem (OTC) has recently been proposed as a closed system for ovarian tissue vitrification [19]. The device material was manufactured from stainless steel, which has a high thermal conductivity and cooling rate. In addition, it reduces handling by the operator, improving the timing of vitrification solutions exposition and removal [20]. This method has been described for the vitrification of ovarian tissue from goats [20], sheep [19], collared peccaries [21], dogs [22], and domestic cats [23]; however, there are no reports in the literature regarding its use in testicular tissue. Based on this, in this study, the morphological evaluation of seminiferous tubules, cell viability, and apoptotic index was assessed to compare the effects of different vitrification methods (Ovarian Tissue Cryosystem, Straws, and Solid Surface Vitrification) on adult cat testicular cells.

## 2. Materials and Methods

### 2.1. Ethics and Animal Selection

The Research Ethics Committee approved this study (protocol number: 2890040516, Federal University of Pará), and a veterinarian monitored all sample collections to certify the health status of the animals. Five mixed-breed adult cats (*n* = 5) aged 1–5 years and weighing 2.5–4 kg were used in this study. The animals came from private owners residing in the city of Castanhal, state of Pará, Brazil, who wanted to neuter their cats at the Veterinary Hospital of the Federal University of Pará. The use of tests in the experiment was previously authorized by private owners.

Before the surgical procedure, a clinical examination was performed to determine the animal’s health status, confirmed by a complete blood count and biochemical profile and a physical examination of the reproductive tract.

### 2.2. Testicular Biopsies Assessment

The cats underwent open bilateral orchiectomy. After the procedure, the samples were transported to the laboratory in Petri dishes (90 mm) containing approximately 5 mL of saline solution (0.9% NaCl) (Eurofarma, São Paulo, SP, Brazil) pre-exposed to ice (20 °C). Testes were washed with 3 mL of saline solution before being transferred to a new plate containing 3 mL of Roswell Park Memorial Institute 1640 (RPMI-1640; Sigma–Aldrich Chemical Company, Saint Louis, MO, USA medium without phenol red (Sigma–Aldrich Chemical Company, Saint Louis, MO, USA) for manipulation. The epididymis and deferred duct were removed, and the tunics were incised using a scalpel blade (No. 24) to access the parenchyma. All procedures were performed on ice (20 °C).

The tests were segmented longitudinally to obtain 9 mm^3^ fragments. A total of 16 fragments were collected from each test (*n* = 10) for a total of 160 fragments. Fragments were distributed and processed according to the experimental design.

### 2.3. Experimental Design

**Figure 1:**
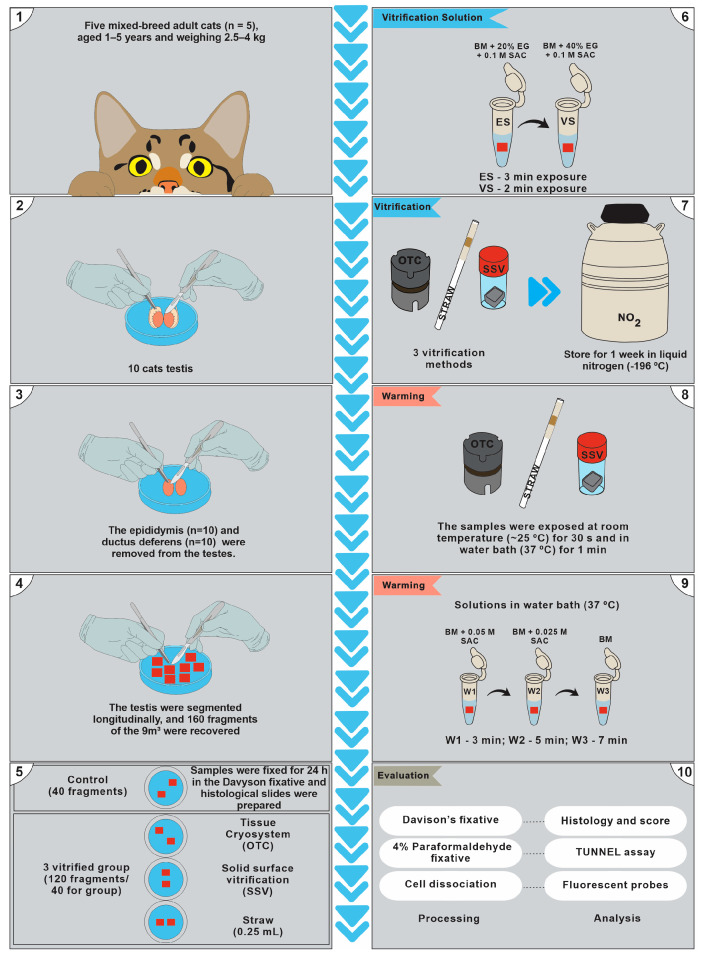
Experimental design. Performance of testis fragment recovery tests (**1**–**4**), separation of experimental groups and control, Ovarian Tissue Cryosystem (OTC), Solid Surface Vitrification (SSV) and Straw (STW) (**5**), exposure of equilibrium solution (ES) and vitrification solution (VS) (**6**), and vitrification process (**7**). Warming device (**8**) and exposure to warming baths (**9**). Testis fragments after warming processing and analysis (**10**).

Cat testicular fragments (*n* = 160) were divided into four groups: control (fresh; *n* = 40) and vitrified samples in OTC (*n* = 40), STW (*n* = 40), and SSV (*n* = 40) (Figure 1). All fragments (fresh control and vitrified) were analyzed using the morphometric scoring scale of seminiferous tubules (histological analysis), cell viability using fluorescent probes (Hoechst-33342 and Propidium iodide; Sigma–Aldrich Chemical Company, Saint Louis, MO, USA), and apoptotic cells using the Terminal deoxynucleotidyl transferase (dUTP) nick end labeling assay (TUNEL; Roche Diagnostics Deutschland GmbH, Mannheim, BD, Germany) (Figure 1).

#### 2.3.1. Vitrification and Warming

**Figure 2:**
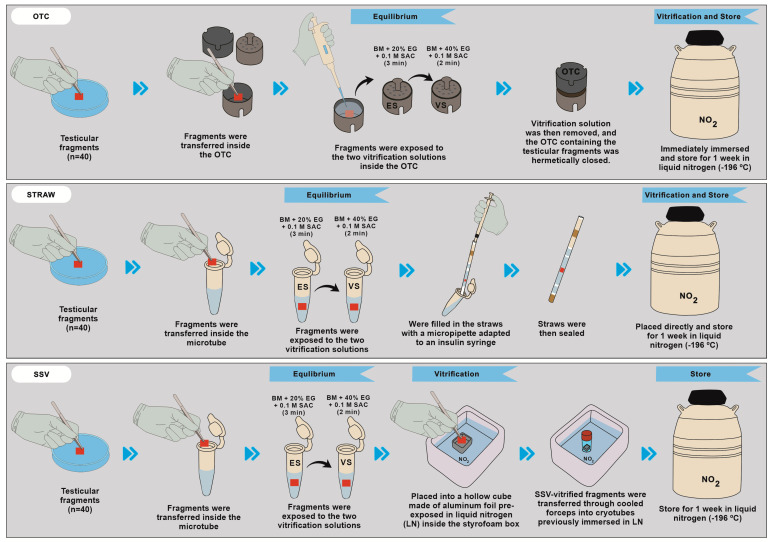
Experimental design demonstrating the three vitrification methods with different stages of exposure and vitrification. Ovarian Tissue Cryosystem (OTC), Straw, and Solid Surface Vitrification (SSV).

Equilibrium and vitrification solutions were prepared just before use. The equilibrium solution was created by adding 20% ethylene glycol (Sigma–Aldrich Chemical Company, Saint Louis, MO, USA) plus 0.1 M sucrose (Sigma–Aldrich Chemical Company, Saint Louis, MO, USA) as a cryoprotectant to RPMI-1640 medium (Sigma–Aldrich Chemical Company, Saint Louis, MO, USA), and the vitrification solution was made by adding Ethylene glycol (40%) plus 0.1 M sucrose. The same cryoprotectant exposition protocol was used for all vitrification methods: the fragments were exposed to the equilibrium solution for 3 min at 20 °C and then to the vitrification solution for 2 min at the same temperature. After the exposure period in equilibrium and vitrification solutions, samples were vitrified using SSV [24], STW [25], or OTC [19] and stored in liquid nitrogen (−196 °C) for one week (Figure 2).

#### 2.3.2. Ovarian Tissue Cryosystem (OTC)

Briefly, the testicular fragments were exposed to the two vitrification solutions inside the OTC. The vitrification solution was then removed, and the OTC containing the testicular fragments was hermetically closed and immediately immersed vertically in liquid nitrogen after storage in liquid nitrogen (−196 °C) [19] (Figure 2).

#### 2.3.3. Straw (STW)

For vitrification in conventional straws (0.5 mm), the testicular fragments were exposed to the vitrification solution in 1.5 mL microtubes; later, they were filled in the straws with a micropipette adapted to an insulin syringe. The straws were then sealed and placed directly in the cryogenic cylinder (Figure 2).

#### 2.3.4. Solid-Surface Vitrification (SSV)

For SSV vitrification, testicular fragments were exposed to equilibrium and vitrification solutions in 1.5 mL microtubes and immediately placed into a hollow cube made of aluminum foil pre-exposed in liquid nitrogen (LN) inside the styrofoam box. SSV-vitrified fragments were transferred through cooled forceps into cryotubes previously immersed in LN and stored in the liquid phase of a liquid nitrogen tank (Figure 2).

#### 2.3.5. Warming

For warming, after a week of storage, the fragments were removed from the cryogenic cylinder and exposed to room temperature (25 °C) for 30 s, followed by 1 min in a water bath (37 °C), until the complete melting of the vitrification solution. Then, the fragments contained in straws and cryotubes were placed in 1.5 mL microtubes and passed through three washing baths using RPMI-1640 medium with decreasing sucrose concentration (0.05, 0.025, and 0 M) for 3, 5, and 7 min, respectively. For the warming, OTC containing the vitrified samples was exposed to room temperature (30 s/25 °C), then OTC was immersed at 37 °C in a water bath for 30 s. After warming of OTC, three washing baths using RPMI-1640 medium with decreasing sucrose concentration (0.05, 0.025, and 0 M) were used for 3, 5, and 7 min, respectively, and were added inside the OTC (Figure 2).

### 2.4. Histological Analysis

Immediately after warming, tissue fragments were prepared for standard histological analyses through a process of dehydration in ethanol, clarification in xylene, and embedding in paraffin. Thin sections (5 µm) of the testicular biopsies were cut, mounted on glass slides, and stained with hematoxylin and eosin. Images of the tissue were captured at 400× magnification using a digital camera coupled with a light microscope and analyzed using NIS Elements^®^ software (v. 5.21, NIS—NIKON, Tokyo, Japan). Thirty tubules from each sample were evaluated for each experimental group. Scoring of spermatogonia nuclei, Sertoli cells, tubular epithelial cell detachment from the basement membrane, and epithelial shrinkage was based on Milazzo et al. [26]. The scoring scale was performed as follows: the nuclei of spermatogonia and Sertoli cells were scored according to (1) distinction between Sertoli cells and spermatogonia nuclei (0 if easy, 1 if difficult, and 2 if impossible); (2) observation of nucleoli (0 if visible in 40% of cells and 1 if indistinguishable in the case of pyknotic nuclei present in a large number and very condensed); and (3) nuclei condensation (0 if absent or present in only 1 nucleus, 1 if present in <40% of nuclei were condensed, and 2 if >40% were pyknotic). The tubular epithelium was scored according to detachment of cells from the basement membrane (0 if absent, 2 if partial, and 3 if present in >75% of the tubule) and epithelial shrinkage (0 if absent, 2 if partial, and 3 if evident).

### 2.5. Cell Viability

Immediately after warming, cell viability was determined using the conjugated fluorescent dyes Hoechst-33342 and propidium iodide (PI) (Sigma–Aldrich Chemical Company, Saint Louis, MO, USA). Testicular cells were mechanically dissociated. Fragments were successively cut in a Petri dish and mixed with 1 mL of RPMI medium. The solution was then transferred to a Falcon tube and pipetted 20 times. Isolated cells were collected using a cell strainer (40 μm). The samples were then microcentrifuged at 1000 rpm for 5 min. A Pellet of a centrifuged solution (150 μL) containing the testicular cells was incubated with 2.5 μL Hoechst-33342 (0.5 μg/mL) and 1 μL PI (2 μg/mL) for 30 min in a dark room [10]. After incubation, 10 μL of the solution was placed on a slide and evaluated using an epifluorescence microscope (Eclipse Ni-U, Nikon Corporation, Tokyo, Japan) at 400× magnification. At least 200 cells per slide were classified as Hoechst + (viable) or PI + (nonviable). The fluorescence signals emitted from propidium iodide and Hoechst were collected at 490/635 nm to Hoechst and 472 nm to PI. The number of Hoechst-positive cells per total number of cells × 100 determined the percentage of viable cells.

### 2.6. Tunel Assay

Immediately after warming, TUNEL assay was performed using the In Situ Cell Death Detection Kit with fluorescein (Roche Diagnostics Deutschland GmbH, Mannheim, BD, Germany) according to the manufacturer’s instructions. After histological processing of the fragments, the 5 µm sections were mounted on slides and submitted to two baths with xylene (5 min each) and 5 baths with decreasing alcohol concentration (100%, 95%, 90%, 80%, 70%) (3 min each), followed by one bath with distilled water. Permeabilization was performed by incubating 50 µL of trypsin (1×) (TrypLE ™ Express, Gibco, ThermoFisher, Carlsbad, CA, USA) for 60 min at 37 °C in a humid chamber. After washing twice with sterile buffered saline (PBS), slides were incubated with a mixture of TUNEL enzyme solution containing terminal deoxynucleotidyl transferase and TUNEL labeling solution containing deoxyuridine triphosphate in a humid chamber at 37 °C for 60 min, followed by three PBS washes. For positive control, slides were incubated with DNase I RNase-free (1 U/µL) (Sigma–Aldrich Chemical Company, Saint Louis, MO, USA) for 30 min before the TUNEL mixture incubation. The sections were counterstained for 10 min using Hoechst 33342 fluorescent probe (0.5 µg/mL) (Sigma–Aldrich Chemical Company, Saint Louis, MO, USA). Each slide was treated with VectaShield^®^ Antifade (Vector Laboratories, Inc., Newark, CA, USA), and a coverslip was placed on top. Negative and positive controls were used for all animals.

The slides were analyzed using an epifluorescence microscope (Eclipse Ni-U; Nikon Corporation, Tokyo, Japan) at 400× magnification. In each experimental group, 50 seminiferous tubules were examined using DAPI (4′,6-diamidino-2-phenylindole; 359/457 nm excitation/emission), FITC (fluorescein isothiocyanate; 495–519 nm excitation/emission), and DAPI/FITC fluorescence filters. Hoechst-positive cells were stained blue, and TUNEL-positive cells were stained green (495/519 nm excitation/emission). The apoptotic index was calculated based on the percentage of TUNEL-positive cells [27].

### 2.7. Statistical Analysis

Data were expressed as mean and standard deviation and analyzed using Statview 5.0 statistical software (SAS Institute Inc., Cary, NC, USA). All data were subjected to the Kolmogorov–Smirnov test and logarithmic transformation. The effects of OTC, STW, and SSV vitrification on the tubular score scale, cell viability, and apoptotic cell index were assessed using analysis of variance, and differences were determined using Fisher’s protected least significant difference post hoc test. *p* < 0.05 was considered statistically significant.

## 3. Results

### 3.1. Histological Analysis

#### 3.1.1. The Distinction between Sertoli Cells and Spermatogonial Nuclei

**Figure 3:**
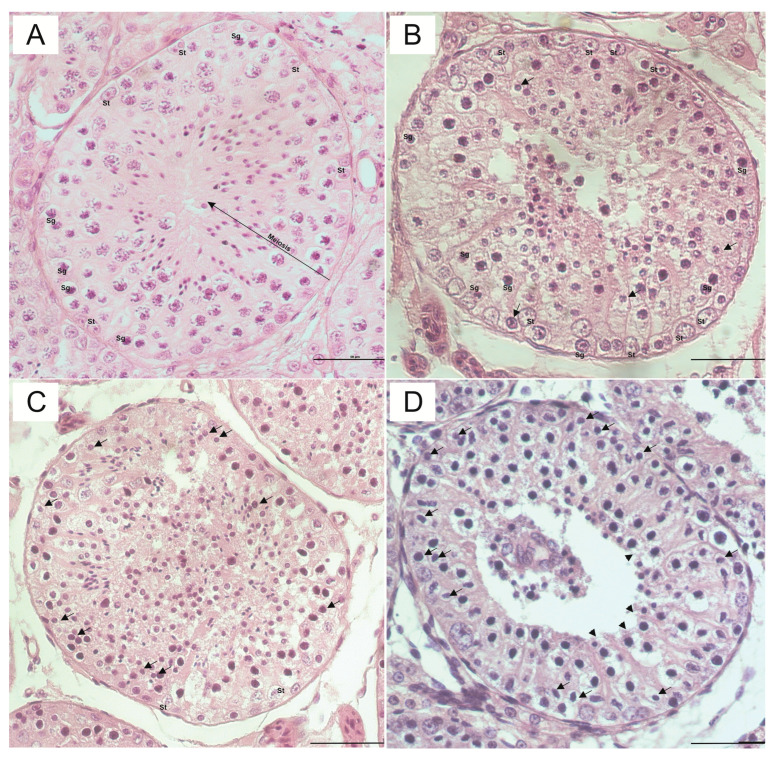
Morphology of seminiferous tubules after cryopreservation with the following conditions (**A**) control; (**B**) OTC; (**C**) Straw, and (**D**) SSV. Scale bar: 50 μm. (**A**) Fresh control: Sertoli cells (small ovoid nuclei arranged perpendicular to the basement membrane; St) and type A spermatogonia (large spherical nuclei; Sg) constitute the seminiferous epithelium. Sertoli cell cytoplasm fills the future lumen of the seminiferous tubules. (**B**) Slightly altered tubule: the nuclei are well preserved (rare pyknotic nuclei; arrow), and the distinction between Sertoli cells and spermatogonia is easy; the epithelium is slightly amended with some gaps. (**C**) Altered tubule: all nuclei are rounded, and the distinction between Sertoli cells and spermatogonia is not possible, except for some cells; a moderate increase in the proportion of pyknotic (arrow) nuclei is observed, and the epithelium is strongly altered with large gaps in the location of the future lumen. (**D**) Severely altered tubule: the distinction between Sertoli cells and spermatogonia is impossible with a large increase in the proportion of pyknotic nuclei and shrinkage of the epithelium (arrowhead).

According to the experiment, in the control group, the nuclei of spermatogonia (large spherical nuclei) and Sertoli cells (small ovoid nuclei arranged perpendicularly to the basal membrane) were well preserved. The distinction between these cells was generally easy to identify since they presented a score of 0.03 on the scale by [26]; this group is significantly different from all vitrified groups (*p* = 0.0025 OTC; *p* = 0.0001 STW and *p* = 0.0001 SSV) (Table 1; Figure 3).

The OTC group was the vitrification method that demonstrated better tissue preservation of the spermatogonial nuclei and Sertoli cells since it obtained a score of 0.6, still being considered as a score of easy cell distinction compared to the other vitrification groups, which presented 1.16 for STW (*p* = 0.0058), and 1.2 for SSV (*p* = 0.0038) group, which was considered significantly difficult to distinguish due to the higher rounding of Sertoli cell nuclei, which made it difficult to differentiate between intratubular cells (Sertoli cells and spermatogonia) at the time of evaluation (Table 1; Figure 2).

#### 3.1.2. Nucleolar Visualization

Regarding nucleolar visualization, the control group showed a better histological appearance since the nucleoli were easily visible in 40% of the cells ( score of 0), being different from the vitrified ones (*p* = 0.0001 OTC, *p* = 0.0001 STW, and *p* = 0.0001 SSV). However, in the STW group, this visualization was difficult, being considered indistinguishable, as it presented a score of 1, where it was possible to observe pyknotic nuclei present in large numbers, being different from the OTC and SSV groups (*p* = 0.01 and *p* = 0.001, respectively). In the OTC and SSV groups, the visualization of nucleoli was considered with moderate alterations in 40% of the cells, with scores of 0.6 and 0.5, respectively (Table 1; Figure 3).

#### 3.1.3. Nuclear Condensation

The absence of nuclear condensation in the control group presented a score closer to zero (0.2), which is considered the best for the classification of nuclear condensation, with the control group being significantly different from all the vitrified groups (*p* = 0.006 OTC, *p* = 0.0004 STW, and *p* = 0.003 SSV). However, moderate nuclear alterations were demonstrated among the vitrified groups, corresponding to less than 40% of the condensed nuclei since they obtained scores close to 1 (Table 1; Figure 3).

#### 3.1.4. Detachment of the Basement Membrane

Although severe cryo-lesions are generally characterized by a large detachment of the basal compartment from the tubular epithelium, in our experiment, the detachment of the epithelium (consisting of intratubular cells) from the cells of the basement membrane was absent in all the groups evaluated, since all the scores are less than or equal to 1, which may demonstrate the preservation of the tubular structure in the different vitrified groups, preserving the architecture of the tubules. However, statistically, there was a difference between the control group and the STW (*p* = 0.03) and SSV groups (*p* = 0.01). It is worth mentioning that the vitrified groups were similar regarding the detachment of the basement membrane from the tubular epithelium (Table 1; Figure 3).

#### 3.1.5. Shrinkage of the Epithelium

In the control and SSV groups, no evident alteration was observed in the shrinkage of the tubular epithelium since the scores were 0.3 and 1, respectively. Nevertheless, although the control group and SSV in the scale are characterized by the absence of epithelium shrinkage, in this parameter, statistically, the control group was significantly higher than all vitrified groups (*p* = 0.0001 OTC, *p* = 0.0001 STW, and *p* = 0.01 SSV). Minor tissue alterations of partial shrinkage of the cytoplasm of some tubular epithelium cells were observed in the OTC and STW groups. They were classified within score 2, and none of the vitrified groups had evident total retraction of the epithelium, preserving the structure of the tubule, and having highlights for the SSV group (Table 1; Figure 3).

In the present experiment, it was demonstrated that among the five evaluations made by histological analysis, the OTC method was the one that showed the best results, followed by the SSV.

### 3.2. Cell Viability

**Figure 4:**
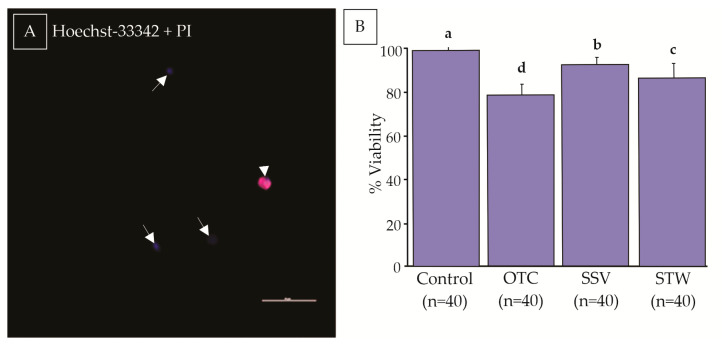
Evaluation of cell viability assessment by Hoechst-33342 and Propidium Iodide (PI) fluorescent dyes. Blue cells were considered viable (arrow), and red cells were nonviable (arrowhead) (**A**). The percentage of cell viability before (control *n* = 40) and after vitrification with Ovarian Tissue Cryosystem (OTC *n* = 40), Solid Surface Vitrification (SSV *n* = 40), or Straw (STW *n* = 40) (**B**). The scale bar represents 50 μm. (a–d) Different letters indicate significant statistical differences among treatments (*p* < 0.05).

The cell viability of the fresh control was superior to that of the vitrified groups (99 ± 0.8) (*p* = 0.0001 OTC, *p* = 0.0248 SSV, and *p* = 0.0002 STW). The percentage of Hoechst-positive cells (viable) was higher in the SSV methods (92 ± 3.3) compared to the STW (86 ± 6.1; *p* = 0.0334) and OTC (79 ± 4.6, *p* = 0.0001) methods. In addition, STW methods had a higher percentage of viable cells when compared to OTC (*p* = 0.00115) (Figure 4). Notably, although the three methods were different regarding plasma membrane viability, they were efficient in the vitrification of testicular tissue from domestic cats, as they showed viability above 70%.

### 3.3. TUNEL Assay

**Figure 5:**
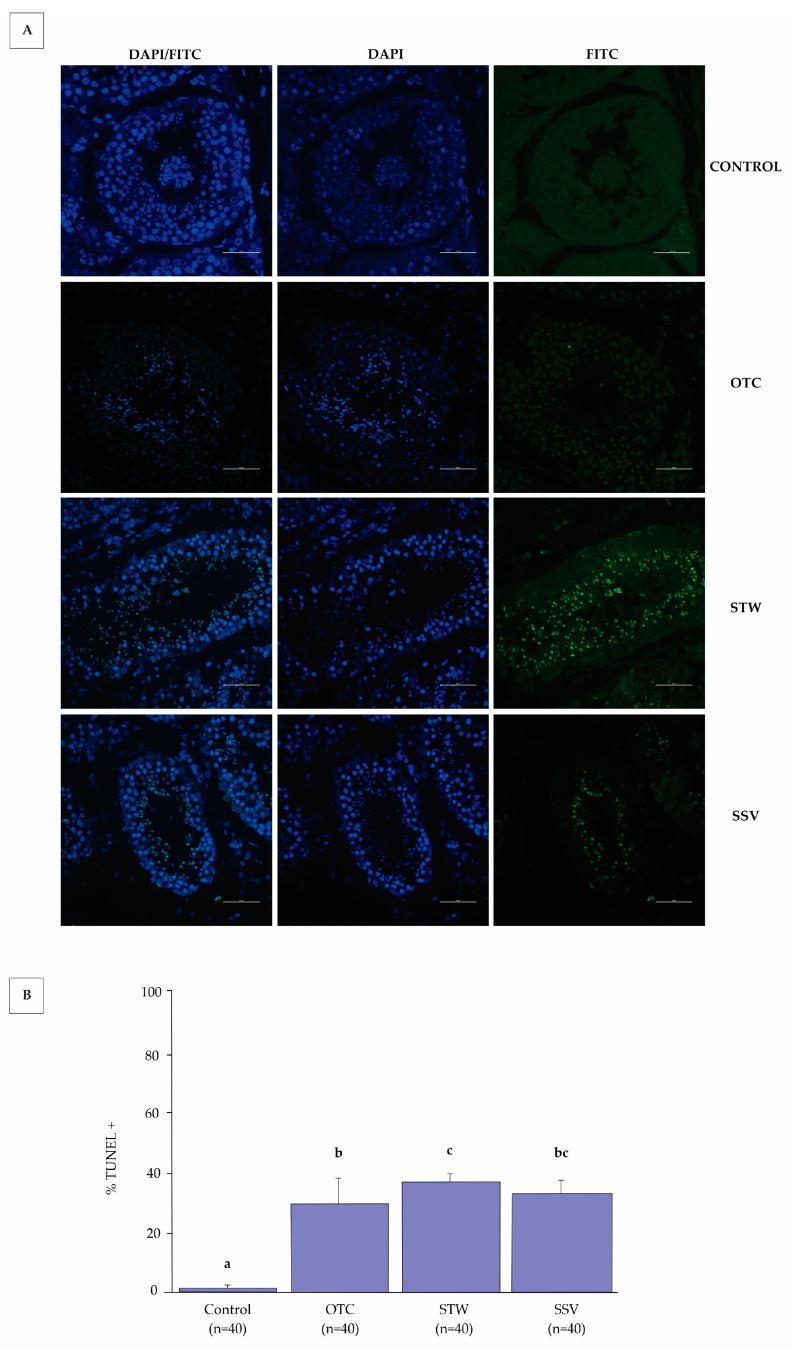
Fluorescein TUNEL assay of cat seminiferous tubules before and after vitrification by Ovarian Tissue Cryosystem (OTC *n* = 40), Straw (STW *n* = 40), or Solid Surface Vitrification (SSV *n* = 40). DAPI: 4,6-diamidino-2-phenylindole; FITC: fluorescein isothiocyanate indicates fluorescence filters used. The Hoechst-positive tubular cells were stained blue, and TUNEL-positive cells were stained green (**A**). The graphic shows the percentage of apoptotic cells (TUNEL-positive) before (control *n* = 40) and after vitrification (**B**). 400× magnification. The scale bar represents 50 μm. (a–c) Different letters indicate significant differences among treatments (*p* < 0.05).

Prior to vitrification (fresh control), cat testicular tubules were minimally affected by apoptosis (2 ± 0.8) (*p* = 0.0001 OTC, *p* = 0.0001 STW, and *p* = 0.0001 SSV). The vitrification device, STW (37 ± 2.8), resulted in a higher apoptosis index than the OTC (29 ± 9.1, *p* = 0.0321). On the other hand, SSV (33 ± 4.3) was statistically equal to the OTC (*p* = 0.2462) and STW (*p* = 0.2696) methods (Figure 5). So, although the three methods were different when evaluating the DNA’s viability, they were efficient since they presented above 60% of DNA integrity (no fragmentation).

## 4. Discussion

This study was able to demonstrate the efficiency of using OTC, SSV, and STW methods and devices for testicular tissue vitrification in domestic cats, regardless of the filling method used, since they presented high cell viability rates, different from what was described by Liebermann et al. who stated that the device used for the vitrification of biological materials could directly influence the result and post-warming cell viability, being considered crucial for cell recovery and survival rates after the vitrification device is used [28]. In this study, all devices used and their vitrification methods effectively preserved the spermatogenic lineage of adult cats in terms of their typical histological aspects.

As verified through histological analysis, after vitrification, nucleolar visualization of spermatogonia and Sertoli cells were less affected by OTC and SSV than plastic straws. This morphological parameter was still scored considerably when prepubertal cat testicular biopsies were vitrified using the SSV method [10]. However, no reports have been on using OTC vitrification for testicular tissues. For domestic cats, a comparison of the OTC and SSV vitrification systems in cat ovarian tissue did not show differences between the techniques in terms of the percentage of morphologically normal follicles after vitrification [29]. However, the differences in cellular metabolism, structure, and endogenous influences between females and males highlight the importance of testicular tissue studies.

It is noteworthy that all vitrification methods prevented basement membrane detachment. However, the SSV method better preserved the tubular epithelium. Changes such as cell separation and shrinkage of the basement membrane can reduce the viability and proliferative capacity of testicular germ cells after cell culture [1], which is evidence of the potential positive effects of these methods. The success of SSV systems for testicular cell preservation has been widely described. Recent studies on goats have shown that this method positively affects isolated Sertoli cells [30] and spermatogonia stem cells [31] regarding the viability and proliferation potential of SSV-vitrified cells.

Although adult testicular biopsies pose some difficulties in preserving tissue architecture, cell–cell communication, and avoiding other mechanical damage, such as microfractures and dryness [32], our results suggest that the SSV method is efficient in preserving the morphological cell structure and viability of adult cat testicular cells. Notably, regarding plasma membrane viability and DNA integrity, the three methods used in our study were above 70% and 60%, respectively.

The conductance of the material that constituted the devices and the vitrification methods used may have directly influenced the results obtained since, unlike device systems such as Straws, the SSV method eliminates the insulating layer between the liquid nitrogen and the vitrification solution caused by the plastic walls of the flasks, which results in high-velocity cooling rates due to the higher conductance of the device (aluminum) [33,34]. High-conductivity materials are essential to avoid apoptosis in ovarian tissue vitrification from OTC [21]. This study was the first to report using OTC for testicular tissue vitrification, which made it very difficult to discuss the findings of the results. Considering that the OTC is a device made of stainless steel, which ensures rapid heat exchange, accelerating temperature reduction, and subsequent heating of the vitrified material [22], these characteristics could minimize the harmful effects and apoptosis occurring after vitrification. Apoptosis is a genetically programmed cell death process that occurs naturally to control cellular multiplication in highly mitotic tissues such as gonads [35]. Therefore, in cases of cold stress, apoptosis may be exacerbated, leading to the absence of viable cells after warming [36].

In SSV, despite the aluminum material of the boats having better conductance than the stainless steel of the OTC and the polypropylene of the plastic straws, the method presents the exposure to cryoprotectants and the vitrification solution and the storage of the fragments in different stages during the processing, making it more difficult to standardize the procedures performed, making it difficult to handle the vitrified boat submerged in liquid nitrogen, which obliges to remove it in liquid nitrogen during the vitrification process. Temperature variations may occur during the glazing process, leading to unwanted glazing/devitrification between steps, somewhat hampering the whole process. In addition, the SSV device comprises an open system with direct contact with liquid nitrogen, making the process more critical for wild animals due to biosafety issues, mainly for wild animals, which generally harbor infectious diseases [37]. In this way, NL filtration has been performed to avoid these problems [38]. The method OTC despite being a device made of material inferior to the SSV in terms of conductance, has the advantage of being a closed vitrification and storage system [19], where the entire exposure procedure to the vitrification solutions is carried out within the storage device without significant temperature variations and without direct tissue contact with nitrogen vapor, which makes it safer against cryogenic resistant pathogens [39]. The closed system showed a lower apoptotic index in tubular cells than the vitrification in the straw [35]. On the other hand, this device occupies more space for storage in the liquid nitrogen cylinder, unlike SSV and STW.

Although plastic straws showed the highest number of apoptotic cells than OTC and SSV, a recent study showed fewer caspase-positive testicular cells in adult cats when the Cryotop system was tested [12], like plastic in the lower conductivity material. Additionally, the direct and permanent contact of testicular tissue with the vitrification solution during the storage period can increase the toxic effects on cells because of the high concentration of the medium by cryoprotectant agents [40].

## 5. Conclusions

Vitrification devices can have a significant effect on cat testicular tissue. However, few works have been developed using different devices and methods for the vitrification of testicular fragments in adult cats. The variability of cells in the testis is a critical factor for successful vitrification, and the conductivity of the material used in vitrification can influence the technique’s effectiveness. Although all tested devices showed morphological preservation, high cell viability, and reduced apoptotic index, SSV and OTC devices were the most efficient in maintaining adult cat testicular integrity/viability after vitrification. This information is valuable for establishing efficient protocols that provide higher survival rates of spermatogenic cells, aiming at the resumption of reproductive functionality and subsequent use of other reproduction biotechnologies and providing bases for studies in wild felines in some degree of threat of extinction.

## Figures and Tables

**Table 1:** Score scale of seminiferous tubules after vitrification by Ovarian Tissue Cryosystem (OTC *n* = 40), Straw (STW *n* = 40), or Solid Surface Vitrification (SSV *n* = 40). Data are expressed as mean ± SD.

	Control	OTC	STW	SSV
Spermatogonia/Sertoli nuclei distinction	0.03 (±0.03) ^a^	0.6 (±0.12) ^b^	1.16 (±0.05) ^c^	1.2 (±0.19) ^c^
Nucleolar visualization	0 (±0.00) ^a^	0.6 (±0.53) ^b^	1 (±0.03) ^c^	0.5 (±0.14) ^b^
Nuclear condensation	0.2 (±0.08) ^a^	0.8 (±0.06) ^b^	1 (±0.00) ^b^	0.8 (±0.21) ^b^
Basal membrane detachment	0 (±0.00) ^a^	0.5 (±0.08) ^ab^	0.9 (±0.17) ^b^	1 (±0.49) ^b^
Shrinkage of epithelium	0.3 (±0.11) ^a^	2 (±0.00) ^c^	2 (±0.09) ^c^	1 (±0.35) ^b^

^a–c^ Different letters indicate significant differences among treatments (*p* < 0.05). Scoring scale: the distinction between Sertoli cells and spermatogonia nuclei: 0—if easy, 1—if difficult, and 2—if impossible; observation of nucleoli: 0—if visible in 40% of cells and 1—if indistinguishable in the case of pyknotic nuclei present in a large number and very condensed; nuclei condensation: 0—if absent or present in only one nucleus: 1—if present in <40% of nuclei were condensed, and 2—if >40% were pyknotic; detachment of cells from the basement membrane: 0—if absent, 2—if partial, and 3—if present in >75% of the tubule; epithelial shrinkage: 0—if absent, 2—if partial, and 3—if evident.

## Data Availability

Data is available on request from the authors.
